# Exercise-Associated Hyponatremia during the Olympus Marathon Ultra-Endurance Trail Run

**DOI:** 10.3390/nu12040997

**Published:** 2020-04-03

**Authors:** Giannis Arnaoutis, Costas A. Anastasiou, HyunGyu Suh, Maria Maraki, Yiannis Tsekouras, Emmanouel Dimitroulis, Marcos Echegaray, Dimitra Papamichalopoulou, Spyridon Methenitis, Labros S. Sidossis, Stavros A. Kavouras

**Affiliations:** 1 Department of Nutrition and Dietetics, Harokopio University, 17671 Athens, Greece; garn@hua.gr (G.A.); acostas@hua.gr (C.A.A.); ytse@hua.gr (Y.T.); emdimitroulis@gmail.com (E.D.); kospapana@yahoo.gr (D.P.); 2 Hydration Science Lab, College of Health Solutions, Arizona State University, Phoenix, AZ 85004, USA; hsuh5@asu.edu; 3 School of Physical Education and Sport Science, National and Kapodistrian University of Athens, 17237 Athens, Greece; mmaraki@phed.uoa.gr (M.M.); smetheni@phed.uoa.gr (S.M.); 4 Department of Biology, University of Puerto Rico at Cayey, 00736 Cayey, Puerto Rico; marcos.echegaray@upr.edu; 5 Department of Kinesiology and Health, Rutgers University, New Brunswick, NJ 08901-8525, USA; lss133@rci.rutgers.edu

**Keywords:** fluid balance, dehydration, underhydration, electrolyte disorders, water intake

## Abstract

Research on hyponatremia during mountain marathons is scarce. The present study aimed to investigate the prevalence of exercise-associated hyponatremia during a 44-km trail running race that reached an altitude of 2780 m (Olympus Marathon). Sixty-two runners (five women) who completed the race participated in the study (age: 34.4 ± 8.6 years; height: 1.77 ± 0.06 m; and weight: 75.3 ± 10.0 kg). Anthropometric characteristics, blood, and urine samples were collected pre- and post-race. Food and fluid intake were recorded at each checkpoint. Due to race regulations, the runners could not carry any additional food and fluids besides the ones provided at specific checkpoints. Five runners (8%) exhibited asymptomatic hyponatremia (serum sodium <135 mmol∙L^−1^). Serum sodium in the hyponatremic runners decreased from 138.4 ± 0.9 (pre) to 131.4 ± 5.0 mmol∙L^−1^ (post), *p* < 0.05. Plasma osmolality increased only in the eunatremic runners (pre: 290 ± 3; post: 295 ± 6 mmol∙kg^−1^; *p* < 0.05). Plasma volume decreased more in the hyponatremic compared to eunatremic runners (−4.4 ± 2.0 vs. −3.2 ± 1.4%, *p* < 0.05). Lastly, dietary sodium intake was lower in the hyponatremic runners compared to eunatremic (789 ± 813 vs. 906 ± 672 mg; *p* < 0.05). The incidence of hyponatremia among the athletes was relatively low, possibly due to race conditions.

## 1. Introduction

Marathon running and ultra-endurance events have gained significant popularity during the last decades worldwide [1,2]. Exercise associated hyponatremia (EAH) is a potentially life-threatening condition that can happen in athletic endurance events lasting more than four hours. EAH is defined as a serum sodium concentration of less than 135 mmol/L during or up to 24 h after prolonged physical activity [3,4] and is mainly caused by excessive sodium loss via sweating and/or excessive drinking of hypotonic fluids [5]. A high incidence of EAH has been documented mainly in ultra-endurance sports events but also in sports of shorter duration such as marathon [6,7,8,9,10]. Exercise-associate hyponatremia has been observed among marathon runners, cyclists, triathlon athletes, and recreational runners, participating most commonly in endurance events [3,4]. Data from studies carried out at the London and Boston Marathons indicate that EAH occurs in 10–15% of sampled marathon finishers [6,8,11].

EAH is predominantly dilutional hyponatremia caused by an increase in total body water relative to the amount of total body exchangeable sodium [3,4,12]. If not promptly recognized and adequately treated, EAH can be fatal via cerebral and/or noncardiogenic pulmonary edema. However, exercise-associated hyponatremia is still underestimated and, therefore, remains a medically significant problem for marathon runners. Moreover, educational interventions towards appropriate fluid intake along with information about EAH’s etiology and symptoms seem essential to reduce the risk and eventually, the incidence of EAH.

Thus, the purpose of the present study was to investigate the prevalence of exercise-associated hyponatremia during a 44-km ultra-marathon mountain trail running race and examine the role of dietary sodium intake.

## 2. Materials and Methods

### 2.1. Subjects

Sixty-two (five women) runners that successfully completed the race participated in the study. The inclusion criteria of the current study were: (1) age over 18 years old; (2) absence of orthopedic/neuromuscular injuries; and (3) previous experience with trail running races. The anthropometrical characteristics of the runners are presented in Table 1.

Participants were informed about the nature and purpose of the study and gave written informed consent. All procedures were in accordance with the Declaration of Helsinki, and the protocol was approved by the institutional review board of Harokopio University. Anthropometric characteristics, blood, and urine samples were collected and evaluated pre- and immediately post-race. In addition, nutritional behavior—fluid and nutrient consumption—was assessed throughout the race.

### 2.2. Race Description

The study was conducted during the official Olympus Marathon race, which is the most prominent mountain race in Greece. Runners covered 44 km on Olympus Mountain, of which 38 km are on mountain trails and 6 km on paved roads. The race started at 3 m above sea-level, reached a maximum altitude of 2780 m and finished at 300 m above sea-level. The winner of the race usually completes the marathon in less than 4:20 h, with a 10 h finishing time limit. The organizing committee of the race is exclusively responsible for providing specific foods and drinks for the athletes, supporting them in 11 intermediate checkpoint stations throughout the mountain, approximately every 3–5 km. Athletes are not allowed to carry any other supplemental foods. The race is designated as an environmentally friendly race since it takes place on the Olympus Mountain, which is one of the Greek National Parks, also listed as a “World Natural Heritage Monument” by UNESCO (United Nations Educational, Scientific, and Cultural Organization). Dropping trash along the race is strictly prohibited and leads to disqualification. Mean environmental temperature and relative humidity during the day of the event ranged 15–34 °C and 50–70%, respectively.

### 2.3. Dietary Intake

Two to three members of the research team were assigned to each of the checkpoints to record dietary intake during the race. The data were collected by audio-recording with the members of the research team mentioning the precise amount and type of fluids and foods that the runners consumed. One size of cups were used in all checkpoints, and food availability was identical in all stations and provided by the race organizers. Since athletes were not allowed to carry and consume any drink or food besides the specific checkpoints, any food and fluid consumed in the 11 checkpoints represented the total amount consumed in the whole race.

### 2.4. Anthropometric Characteristics

The day before the race, height was measured using a stadiometer with an accuracy of 0.5 cm (SECA 220, Seca Corporation, Columbia, USA), and body weight was recorded to the closest 100 g (Seca, model: 7701321004, Vogel & Hamburg, Germany). Body mass index (BMI) was calculated as body weight in kilograms, divided by the square of height in meters (kg/m^2^).

### 2.5. Blood and Urine Analysis

Blood samples were collected from runners following 15 min seated rest and analyzed immediately in duplicate for hematocrit (microhematocrit method) and hemoglobin (cyanmethemoglobin method, Drabkin reagent; Sigma-Aldrich, St. Louis, MO, USA), and the Dill and Costill equation was used to calculate changes in plasma volume [13]. In the remaining blood, serum and plasma were separated from the blood cells by centrifugation. Aliquots of blood plasma were used fresh for the determination of plasma osmolality by freezing-point depression (3D3 Osmometer; Advanced Instruments Inc., Norwood, MA, USA). The remaining blood plasma and serum were stored frozen (−80 °C) for subsequent analysis of serum glucose and creatine kinase serum sodium and potassium. Glucose and creatine kinase were measured by enzymatic analysis in an automated biochemical analyzer (ACE; Schiapparelli Biosystems Inc., Fairfield, NJ, USA). Electrolytes concentration was measured by selective electrode conductivity in an automated analyzer (Ektachem DT60 II system; Eastman Kodak Co., Rochester, NY, USA). Urine osmolality was measured in duplicate by freezing point depression (3D3 Advanced Osmometer, Advanced Instruments Inc., MA, USA) and specific gravity (USG) with a handheld refractometer (ATAGO SUR-NE, Tokyo, Japan).

### 2.6. Statistical Analysis

All data are presented as means ± one standard deviation. Student t-test was performed to compare differences between groups and paired t-test for pre- to post-race differences. Effect size was calculated via partial eta square. Statistical analyses were performed with JMP Pro 14 (SAS Institute Inc., NC, USA). Statistical significance was accepted at *p* ≤ 0.05 for all tests.

## 3. Results

Of the 62 volunteer athletes who participated in the study, five (8%) experienced exercise-associated hyponatremia (serum sodium concentration of <135 mmol∙L^−1^). Only one of those five runners experienced severe hyponatremia (<130 mmol∙L^−1^) with sodium levels of 122 mmol∙L^−1^.

Mean body weight decreased in both eunatremic and hyponatremic group, but the difference between the two groups was not statistically significant (*p* ≥ 0.05, Table 2). Hyponatremic runners experienced a greater percentage of body weight loss than the eunatremic ones (*p* < 0.05, Table 2). However, there were no significant differences in finishing time and total fluid intake between both groups. Hypovolemia after the race, as indicated by lower plasma volume, was greater in the group with hyponatremia compared to the eunatremic one (*p* < 0.05).

**Figure 1:**
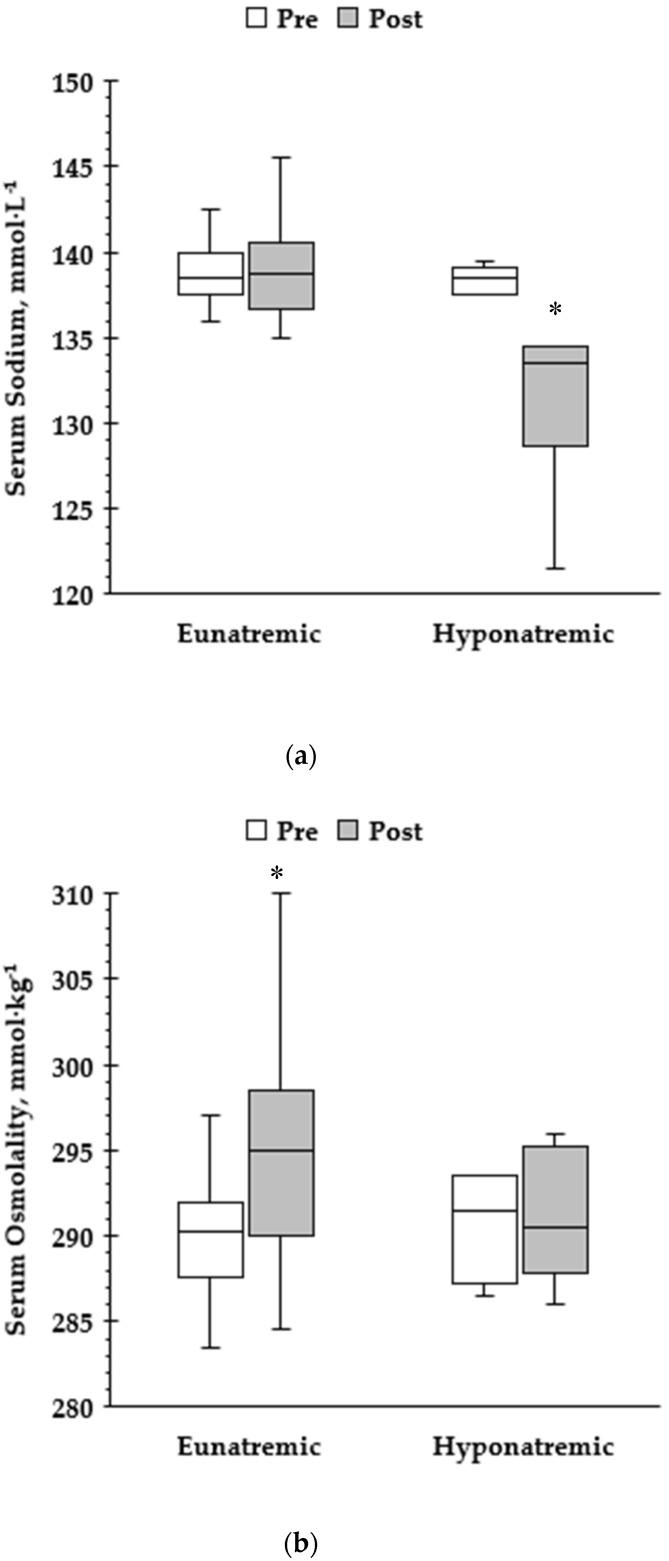
Serum sodium (**a**) and serum osmolality (**b**) level (Pre vs. Post) in eunatremic vs. hyponatremic runners. * Denotes statistically significant difference from baseline value.

Serum sodium decreased in the hyponatremia group (pre: 138.4 ± 0.9; post: 131.4 ± 5.0 mmol∙L^−1^; *p* < 0.05), while it was maintained in the eunatremic group (pre: 138.7 ± 1.9; post: 139.2 ± 3.4 mmol∙L^−1^; *p* ≥ 0.05; Figure 1). Eunatremic runners had higher serum osmolality after the race (pre: 290 ± 3; post: 295 ± 6 mmol∙kg^−1^; *p* < 0.05), while no differences were observed in the hyponatremic runners (pre: 291 ± 3; post: 291 ± 4 mmol∙kg^−1^; *p* ≥ 0.05; Figure 1). On the contrary, serum potassium significantly decreased in the eunatremic group (*p* < 0.05), while it was maintained in the hyponatremic group (*p* ≥ 0.05; Table 2). Serum creatine kinase activation was significantly increased post-race (*p* < 0.05) for both groups with the hyponatremic athletes exhibiting higher values, while serum glucose remained unaffected (*p* ≥ 0.05) in both eunatremic and hyponatremic group.

There was no significant change in urine osmolality between pre- and post-race for both eunatremic and hyponatremic groups (*p* ≥ 0.05; Table 2). However, USG significantly increased after the race only in the eunatremic group (*p* < 0.05). A significant decrease was observed in urine sodium post-race (*p* < 0.05), while the increment was observed in both eunatremic and hyponatremic group for urine potassium (*p* < 0.05). However, no statistical difference was observed between the two groups for urine sodium and urine potassium (*p* ≥ 0.05). Potassium-to-sodium ratio in urine was increased in both eunatremic and hyponatremic runners (*p* < 0.05), and the increment was higher in the hyponatremic runners (*p* < 0.05).

The results from the dietary intake during exercise are presented in Table 3. Dietary sodium intake during the race was significantly lower in the hyponatremic versus the eunatremic runners (*p* < 0.05), while there was no statistical difference in other nutrient intake (*p* ≥ 0.05). There was no significant difference in fluid intake between two groups (*p* ≥ 0.05).

## 4. Discussion

In the present study, we examined the prevalence of exercise-associated hyponatremia in a sample of runners participating in the Olympus Marathon. The incidence of mild and moderate hyponatremia was 8%, close to that observed in the previous studies mentioned above. The decrease in serum sodium in the hyponatremic group did not lead to lower serum osmolality. Interestingly, the increase in serum osmolality after the race in the eunatremic runners was not linked to changes in serum sodium. Changes in serum sodium and osmolality are tightly regulated in the body in order to maintain body fluid homeostasis, and multiple factors could contribute to these adjustments, especially during the race, such as finish time and fluid intake. No significant difference in serum sodium concentration was observed in the eunatremic runners, yet serum osmolality increased by 5 mmol·kg^−1^, possibly due to changes in concentration in other osmotically active substances that we did not measure. However, it is unclear what led to this response in the current study.

Body fluids need to be replenished to prevent athletes from excessive dehydration during exercise [12]. However, fluid overload is a dangerous practice during prolonged exercise, and it should be avoided [14]. The amount of consumed fluid can vary among individual runners to compensate body fluid loss [15]. Excessive fluid drinking has been reported to be one of the factors that could cause EAH [16]. In the present study, no statistical differences were found in total fluid intake between eunatremic and hyponatremic groups. Exercise duration can also be a contributing factor in the development of EAH [6]. However, in the current data, no significant difference in finishing time was found between eunatremic and hyponatremic runners.

Exercise induced dehydration often leads to hypovolemia as a response to the fluid deficit and exercise itself [17]. In the present study, even though the percent of body mass loss was greater in the hyponatremic group, no difference was observed in the degree of hypovolemia. Presumably, given the race duration, a reasonably large component of the body weight loss could be attributed to glycogen loss and its associated water decrement. It has also been suggested that a decrease in serum potassium is associated with rhabdomyolysis in ultra-endurance runners, but whether this is prominent in the people with EAH remains controversial [18]. Although the decrement of serum potassium was less in the group with hyponatremia in the current study, the increment of serum creatine kinase was rather higher in this group (above 1000 U∙L^−1^), indicating significant muscle damage. Moreover, the significant increase observed in urinary K^+^/Na^+^ ratio, especially in the hyponatremic group, suggests a parallel increased aldosterone activity, possibly due to endocrine-induced renal water retention, in order to maintain blood flow during prolonged exercise.

High carbohydrate consumption is prevalent in endurance events lasting more than one hour. Exogenous carbohydrate intake during the race could maintain a high carbohydrate oxidation rate, sustaining the runner’s pace, and maintaining euglycemia [19,20]. The guidelines from the joint position stand about nutrition and athletic performance suggest that during, endurance events longer than 2.5–3 h, the consumption of carbohydrates should be up to 90 g/h mainly through the provision of multiple transportable carbohydrates in order to maximize oxidation rate [19]. However, the relevant consumption of the runners in the present study was 31 ± 14 and 25 ± 10 g/h for the eunatremic and hyponatremic group, respectively, far below the aforementioned suggestions. Even though competitive ultra-endurance athletes may be more aware of their dietary sodium intake to reduce the risk of EAH [21], sodium intake level in the Olympus Marathon runners was small given the duration of the event [19]. Interestingly, the amount of sodium consumed by the runners that developed hyponatremia was significantly lower than that consumed by the eunatremic runners. These findings, in combination, are in accordance with those from other studies, which clearly indicate that athletes fail to meet the suggested dietary needs, reducing consequently their potential for optimal performance [22,23,24].

As mentioned above, the primary etiologic factor of EAH appears to be the overconsumption of water and/or other hypotonic fluids in excess of total body fluid losses. Under these circumstances, inappropriate vasopressin secretion can reduce renal water clearance resulting in fluid overload and dilutional hyponatremia [3,25]. Due to the race regulations, however, the runners could not carry along any extra personal fluids, something that limited fluid availability. Thus, overconsumption of water or any other hypotonic fluids was unlikely to be observed. Even though some scientists argue that “drinking to thirst” is a good way to prevent exercise-associated hyponatremia, the race regulations enforced a drinking pattern more like “prescribed drinking” than “drinking to thirst”. A strength of the present study derives from its assessment of nutritional intake. Most of the studies with endurance athletes use recall diaries in order to estimate nutritional behaviors [20,26,27,28]. However, the results of the questionnaires depend on the ability of the subjects to remember what they ate and drunk in many checkpoints during a multi-hour race. Exhaustion also could hamper athletes’ memory inducing recall bias. Nonetheless, in the present study, due to the race regulations, we are confident that the recorded dietary data closely reflect the amount of fluids and foods consumed by the athletes during the race.

A limitation of the current study is the statistical analysis of the unequal sample size. In the present study, even though 62 participants agreed to participate, the number of hyponatremic runners was small for the statistical analysis performed with small effect size. However, the numbers were too small to report, probably due to unequal sample size. The large number of paired t-tests performed in the absence of any controls may have significantly increased the risk of type 2 error. Furthermore, serum creatine kinase and serum potassium failed on the normality test after Shapiro–Wilk W test. Another limitation was the incapacity, due to reasons of practicality, of collecting any sweat samples, which, in turn, would have provided additional information concerning electrolyte losses.

## 5. Conclusions

The recent study showed that 8% of runners experienced mild to moderate hyponatremia during the Olympus Marathon ultra-endurance trail run, and this incidence rate was similar to that observed in previous studies. In addition, it is highlighted that continuous efforts made by athletes, coaches, and sports nutritionists, towards educating athletes about the detrimental effects of EAH and the importance of adopting realistic nutrition and hydration race strategies, seems essential.

## Figures and Tables

**Table 1:** Characteristics of the study participants.

Characteristics	*n* = 62
Age (years)	34.4 ± 8.6
Sex (men/women)	57/5
Weight (kg)	75.3 ± 10.0
Height (m)	1.77 ± 0.06
BMI (kg/m^2^)	24.2 ± 1.6

**Table 2:** Body mass, blood, and urine indices of pre- and post-race in eunatremic and hyponatremic runners.

	Eunatremic (*n* = 57)		Hyponatremic (*n* = 5)	
	PRE	POST	PRE	POST
Body Mass, kg	75.2 ± 9.1	73.0 ± 9.1	81.9 ± 10.5	80.1 ± 11.6
Change Body Mass, %	-	−2.4 ± 3.2	-	−3.0 ± 2.2 ^†^
Finishing Time, h	-	8.7 ± 1.6	-	9.3 ± 0.5
Hematocrit, %	42.0 ± 4.4	43.9 ± 2.9	39.7 ± 5.2	43.2 ± 5.3
Plasma Volume Change, %	-	−3.2 ± 1.4	-	−4.4 ± 2.0 ^†^
Serum K^+^, mmol∙L^−1^	5.1 ± 0.7	4.7 ± 0.5 *	5.5 ± 1.2	5.2 ± 0.4
Serum Glucose, mg∙dL^−1^	94.1 ± 19.3	86.5 ± 28.7	95.3 ± 21.0	83.9 ± 35.9
Creatine Kinase, U∙L^−1^	162 ± 115	994 ± 772 *	173 ± 98	1,516 ± 1.213 *^,†^
Urine Osmolality, mOsm∙kg^−1^	721 ± 343	768 ± 288	622 ± 341	729 ± 78
Urine Specific Gravity	1.026 ± 0.011	1.034 ± 0.012 *	1.024 ± 0.012	1.032 ± 0.005
Urine Na^+^, mmol∙L^−1^	187 ± 233	72 ± 43 *	152 ± 98	41 ± 19 *
Urine K^+^, mmol∙L^−1^	42 ± 31	77 ± 38 *	30 ± 11	98 ± 36 *
Urine K^+^/Na^+^ Ratio	0.36 ± 0.22	1.38 ± 0.88 *	0.25 ± 0.21	3.02 ± 2.93 *^,†^

* Denotes statistically significant difference from baseline value. † Denotes statistically significant difference between eunatremic and hyponatremic runners.

**Table 3:** Participants’ energy intake, nutritional evaluation of macro- and micronutrient components, and fluids consumption during ultra-endurance race.

	Dietary Intake during Race	
	Eunatremic	Hyponatremic
Energy intake (kcal)	1525 ± 717	1383 ± 699
Carbohydrates (g)	264 ± 122	236 ± 98
Proteins (g)	43.9 ± 32.7	43.5 ± 35.9
Fat (g)	30.9 ± 25.0	27.1 ± 21.5
Sodium (mg)	906 ± 672	789 ± 813 *
Fluids (mL)	3004 ± 1228	2698 ± 812

* Denotes statistically significant difference between eunatremic and hyponatremic runners.

## Dedication

The authors would like to dedicate this paper to Marcos Echegaray, who unexpectantly passed away at Cayey, Puerto Rico on 2 May 2019. He was training for the 2019 Comrade Marathon in South Africa.
